# Short-term alterations of brain network properties in subthreshold depression: the impact of Internet-based Cognitive Behavioral Therapy

**DOI:** 10.3389/fneur.2025.1474339

**Published:** 2025-04-15

**Authors:** Wenquan Yu, Yuchen Ying, Li Wang, YiLing Yang, Li Zhang, Yu Wang, Zili Zhu, Hui Zhang, Yuning Pan

**Affiliations:** ^1^Department of Radiology, The First Affiliated Hospital of Ningbo University, Ningbo, Zhejiang, China; ^2^Ningbo College of Health Sciences, Ningbo, Zhejiang, China

**Keywords:** cognitive behavioral therapy, nodal efficiency, left paracentral lobule, functional magnetic resonance imaging, brain network analysis

## Abstract

**Purpose:**

Patients with subthreshold depression (sD) carry a significant risk of developing major depressive disorder. However, few studies focus on the influence of Internet-based Cognitive Behavioral Therapy (ICBT) on brain network, particularly among Chinese undergraduates. This study aims to conduct resting-state functional MRI (rs-fMRI) to explore the effects of ICBT on neurofunctional network.

**Methods:**

This short-term longitudinal study enrolled 30 sD patients and 24 healthy matched participants. We gathered the clinical measurements outcomes from sD patients. Baseline and post-intervention MRI scans were performed for the sD patients, additionally, a matched healthy controls group underwent baseline MRI scans, functional network matrix was established from the temporal rs-MRI series. Nodal efficiency (*E*nod) was quantified by calculating the area under the curve (AUC) of network metrics.

**Results:**

The patients with sD exhibited reduced clinical measurement scores after ICBT intervention. Moreover, after ICBT intervention, functional network analysis revealed an increased *E*nod in the orbital part of the left inferior frontal gyrus, and decreased *E*nod in the left paracentral lobule and right precentral gyrus.

**Conclusion:**

This study showed alterations in brain network in patients with sD after ICBT intervention. These findings shed light on the neurofunctional mechanisms underlying the effects of ICBT in sD patients.

## 1 Introduction

Subthreshold depression (sD), characterized by the presence of 2 to 4 depressive symptoms lasting more than two weeks, falls short of meeting the comprehensive criteria for Major Depressive Disorder (MDD) as delineated in the Diagnostic and Statistical Manual of Mental Disorders (DSM) (1, 2). Despite not reaching the severity of MDD, sD is a significant mental health concern due to its potential to progress into full-blown depression (3). This condition, is commonly observed across various age groups including university students at pivotal life stages, contributes not only to the individual neuropsychiatric symptomatology but also imposes a considerable socio-economic burden (4–6). The transition from sD to MDD underscores the critical need to elucidate its underlying neurophysiological mechanisms. A comprehensive understanding of these mechanisms is essential for the development and implementation of effective intervention strategies, which may aid in mitigating or even preventing the progression of sD to MDD.

MDD is a severe mental health challenge, persisting for at least two weeks and characterized by sustained sadness, loss of interest in enjoyable activities, and various physical ailments (7). Globally, MDD has a profound impact, affecting individuals in diverse regions with significant variation in prevalence rates, studies indicate a lifetime prevalence of about 16.6% in the United States and 12.8% in several European countries (8, 9). Additionally, another meta-analysis on subthreshold depression in the elderly report prevalence rates of 1.38–26.7% in Europe, 2.3–58.18% in the United States, and 3.5–56.89% in Asia (10). MDD is not limited to personal suffering, also often leads to social dysfunction, quality of life deterioration, and increased suicide risk (11). Furthermore, research suggests that a substantial number of MDD cases are preceded by a period of sD (12). Therefore, it is imperative to promptly identify and intervene in cases of sD to reduce the risk of its progression to MDD.

Cognitive Behavioral Therapy (CBT) has been recognized as an effective intervention for individuals experiencing depression. The efficacy of CBT has been demonstrated in numerous randomized controlled trials (RCTs) (13). However, the conventional face-to-face format of CBT poses limitations such as the need for in-person sessions and associated costs. These limitations are addressed by the development of Internet-based Cognitive Behavioral Therapy (ICBT), which enhances accessibility and convenience (14). Notably, evidence from a meta-analysis has demonstrated the effectiveness of ICBT in alleviating depressive symptoms in adults with subthreshold depression (sD) (15). Another study from a meta-analysis highlighted that ICBT showed enhanced short-term efficacy compared to control group in patients with sD (16).

ICBT provides several advantages over traditional CBT, such as flexibility in scheduling and the potential for anonymity, which can reduce stigma and discomfort associated with seeking mental health treatment (17). ICBT can be cost-effective and easily accessible, thus increasing the reach of mental health services to larger populations, including those in remote or underserved areas (18). As demonstrated in these studies, ICBT is not only a practical adaptation of traditional CBT but also an effective means of delivering therapy, especially for conditions like sD where early intervention is key to preventing the progression to more severe depressive disorders (15, 19, 20).

However, in China, the public health landscape is notably impacted by MDD and sD, with the latter particularly prevalent. Research indicates that the prevalence rates for MDD stand at 3.4% (21), moreover, a study identified a 14.7% prevalence of sD among adults in southern China, highlighting its substantial impact on mental health (22). Moreover, a study indicates that 30–40% of Chinese adolescents experience sD (23). Furthermore, In China, cultural and socio-economic barriers often prevent individuals from seeking conventional face-to-face mental health treatment. Fortunately, ICBT has become a valuable alternative, offering accessible care through a digital format (18, 21). Random clinical trials have affirmed the effectiveness of ICBT in managing sD within the Chinese population (14). Nevertheless, the mechanistic insights into ICBT are still profoundly lacking.

Resting-State Functional Magnetic Resonance Imaging (rs-fMRI) has emerged as a pivotal tool in psychiatric research, offering valuable insights into intrinsic brain activities, which holds extensive applications in numerous neuroimaging studies (24–26). The values of rs-fMRI in elucidating the neural underpinnings of MDD are well-established in psychiatric research (27). Furthermore, studies have noted that CBT treatment influences the Default Mode Network (DMN) in patients with sD (28). However, its application in studying sD treated by ICBT especially among undergraduates is not as extensive (27, 29). In this research, we employ rs-fMRI to explore the neurobiological mechanisms involved in treating sD with ICBT. This investigation is directed toward augmenting the therapeutic efficacy of ICBT by exploring its impact at the neural level.

This study is designed to achieve these objectives: (1) Evaluating the efficacy of ICBT in depression management; (2) Elucidating the neurobiological mechanisms of ICBT’s therapeutic effects via rs-fMRI; (3) Providing empirical evidence to facilitate the widespread clinical implementation of ICBT in mental health care.

## 2 Materials and methods

### 2.1 Research design

This research conducted as a prospective, short-term longitudinal study. Participants were briefed on the examination procedures and methods prior to the commencement of the study. The ICBT group engaged in a four-week, self-administered ICBT program without direct psychiatric supervision. Baseline and post-treatment MRI scans were conducted for this group, supplemented by a suite of behavioral and cognitive evaluations after MRI scans. A gender, age and degree of education matched healthy control (HC) group, was also subjected to MRI scans at baseline. The study participants were thus categorized into three distinct groups: pre-intervention ICBT (pre-ICBT), post- intervention ICBT (post-ICBT), and the HC group. Conducted under the ethical guidelines of the Medical Ethics Committee of Ningbo University (No. NBU-2020-139), the study ensured comprehensive briefing on its objectives and methodologies to each participant, securing written informed consent before commencement.

### 2.2 Participants and recruitment

From December 2021 to June 2023, a comprehensive recruitment process involving telephone, WeChat, and direct interactions was implemented at diverse colleges in Ningbo in Zhejiang Province, China. The sample size was determined using G*Power (version 3.1.9.7), based on a previously reported RCT survey with an effect size of 0.52 (30). With a one-tailed alpha level of 0.05 and modest power (1-beta = 0.8), the minimum required sample size was 25 sD patients. To account for an estimated 20% attrition rate, the final target sample size was set at 30 sD participants. This recruitment process was conducted using an online questionnaire designed to gather demographic information, as well as scores from the Patient Health Questionnaire-9 (PHQ-9) and the Center for Epidemiological Studies Depression Scale (CES-D). All recruits were college undergraduates, ensuring homogeneity in educational background. Inclusion criteria for the ICBT group were: (1) Chinese undergraduates (included junior college students), (2) CES-D scores ≥ 16, indicating sD (31), (3) PHQ-9 score ≥ 9, (4) age above 18 years, and (5) adeptness in using smart devices.

Exclusion criteria were meticulously set to include: (1) PHQ-9 item 9 score above 2, indicative of suicidal inclinations, (2) medical history of neurological or psychiatric conditions, (3) cerebral anomalies like trauma, tumors, or infections, (4) disorders potentially impacting brain functionality such as mania, severe depression, or bipolar disorders, (5) background of substance misuse, (6) alcohol dependency, and (7) contraindications to MRI or inability to undergo the scanning process. Ultimately, this study enrolled 30 participants in the ICBT group (aged 18–20 years, 25 females/5 males) and 24 matched individuals in the HC group (aged 19–22 years, 20 female/4 males).

### 2.3 Clinical intervention

In current study, the ICBT was developed based on the Chinese version of the Cognitive Behavioral Therapy Manual. This program comprising five lessons, included extensive case narratives to complement the CBT skills taught. The ICBT was conducted via a *Healthy Psychological Station* integrated into a WeChat mini-program, spanning five weeks under clinical supervision. Each week focused on a specific CBT technique: emotional awareness and relationship building (Week 1), cognitive restructuring (Week 2), behavioral activation (Week 3), core belief modification (Week 4), and skill consolidation with future planning (Week 5). Lessons were sequentially unlocked, included real-life case stories, and were reinforced through interactive assignments, clinician support, and automated reminders to enhance engagement and adherence. More detailed exposition of the intervention’s structure and methodology, refer to the corresponding articles (14, 32).

### 2.4 Measures

#### 2.4.1 Primary clinical measurements

The Chinese version of the CES-D: The CES-D comprises 20 items, each item assessed on a scale from 0 to 3. The cumulative score of scale can range from 0 to 60, where a higher score signifies an increased likelihood of experiencing depressive symptoms. The reliability and validity have been validated in Chinese populations (33).

#### 2.4.2 Secondary clinical outcome measures

The PHQ-9 and the Chinese version of the Generalized Anxiety Disorder-7 (GAD-7) were utilized to evaluate symptoms of depression and anxiety respectively, and both have exhibited notable reliability and validity in Chinese populations (34–36).

The Cognitive Behavioral Therapy Skills Questionnaire (CBTSQ) and the Competencies of Cognitive Therapy Scale – Therapist Report version (CCTS-TR) was utilized to assess the competence of participants in CBT skills usage, emphasizing the evaluation of both cognitive and behavioral competencies (37, 38). Notably, the CCTS-TR scale assessments were conducted by CBT therapists, who remain blinded to other research elements to ensure objectivity. The Frequency of Actions and Thoughts Scale (FATS) evaluated the frequency of CBT skills usage across four different dimensions (20).

### 2.5 fMRI data acquisition

The fMRI data were collected by utilizing 3.0 T Siemens MAGNETOM Vida (Siemens, USA) with a 64-channel phased-array head coil. Before performing fMRI sequences, the T1WI, T2WI, T2-FLAIR and DWI imaging were collected, these images were evaluated by experienced radiologists to exclude participants with structural brain abnormalities. For each participant, rs-fMRI scan was conducted over approximately 10 min (54 slices, FOV = 216mmx216mm, TR = 2000 ms, TE = 30 ms, flip angle = 90°, matrix = 72 × 72, thickness = 3 mm, voxel = 3x3x3mm^3^, 200 volumes). Participants were positioned supine with their heads immobilized using foam pads, and earplugs were provided to mitigate noise during the rs-fMRI scanning process, meanwhile, the participants were advised to keep awake, remain calm and to avoid thinking about anything specific. Structural 3D-T1 weighted images were obtained by applying a 3D-MPRAGE sequence (192 slices, FOV = 256mmx256mm, TR = 2,300 ms, TE = 2.98 ms, flip angle = 9°, matrix = 256 × 256, thickness = 1 mm, voxel = 1x1x1mm^3^). For the ICBT group, MRI scans were completed within 48 h at baseline and after the ICBT intervention.

### 2.6 fMRI data preprocessing

All rs-fMRI data were preprocessed in Resting-State fMRI Data Processing Toolkit Plus (RESTplus, version 1.27^1^) and Data Processing Assistant for Resting-State fMRI (DPARSF, version7.0^2^), which is based on the Statistical Parametric Mapping (SPM12) (39). The preprocessing for rs-fMRI data was executed through several specialized steps: (1) To facilitate the achievement of steady-state magnetization and to acclimatize participants to the scanning environment, the first 10 time points were discarded, (2) realigned the time series of functional images to the initial frame to correct for any head movements, participants exceeding 3 mm in translation or 3° in rotation being exclude, (3) co-registered with each participant’s high-resolution structural T1 images, followed by segmentation and alignment to a standard brain template based on deformation field mapping, (4) an isotropic Gaussian kernel with a 6 mm full-width at half-maximum (FWHM) was applied for spatial smoothing to reduce noise and improve signal quality, (5) removing linear trends, addressing potential biases introduced by MRI equipment, (6) removal of nuisance covariates, encompassing Friston’s 24-parameter motion model and signals from white matter and cerebrospinal fluid, (7) to eradicate the confounding influences of ultra-low frequency drift, white matter-derived signals, and high-frequency physiological noises, a frequency filter range of 0.01–0.08 Hz was applied.

### 2.7 Cerebral functional network construction and analysis

For functional brain network construction, we formulated a brain network by defining nodes as specific brain regions on account of the Automated Anatomical Labeling (AAL) atlas, which represented a standard in graph theory analysis, resulting in 90 anatomical regions, inclusive of 78 cortical (each hemisphere contains 39 regions) and 12 subcortical areas (each hemisphere contains 6 regions). The nodes represented various brain regions, and the edges symbolized the connections between these nodes. Subsequently, the partial correlation coefficients of average time series from each region were calculated, while controlling the impacts of remaining 88 regions (representing the conditional dependency), thus, for each participant, a comprehensive 90 × 90 partial correlation matrix was created. To facilitate further analysis, according to the predefined sparsity thresholds (0.10–0.34, interval was 0.01), this sparsity thresholds generated sparse networks that accurately captured small-world properties while reducing spurious edges (40), these partial correlation matrices were transformed into binary matrices. Finally, for each subject, neurofunctional networks under specified sparsity threshold, we employed Graph Theoretical Network Analysis Tool Box (GRETNA^3^) for network analysis, and calculated the area under the curve (AUC) of whole network metrics that offered topological properties. Subsequently, we calculated the AUC of small-world properties of the whole-brain topological network at the predefined sparsity threshold, including global efficiency (Eg), clustering coefficient (Cp), shortest path length (Lp), normalized clustering coefficient (*γ*), normalized characteristic path length (*λ*), small-worldness (*σ*), local efficiency (Eloc), nodal betweenness centrality, nodal degree centrality, and nodal efficiency (*E*nod).

*E*nod quantified the capability of a node to disseminate information to another nodes in brain network, serving as a metric for assessing changes in functional connectivity across different brain regions (41). Elevated nodal efficiency indicates a node’s pivotal function in information transfer within the neural network, marking it as a central hub in facilitating efficient communication, and quantitatively measuring the information transformation of neural networks (42).

### 2.8 Statistical analysis

In order to examine the impact of depression on brain functional dynamics, we performed two-sample *T*-tests to evaluate intergroup differences in *E*nod and global brain attributes between the pre-ICBT and HC groups, as well as between the post-ICBT and HC groups. Subsequently, to ascertain the modulatory effects of ICBT on neural connectivity, we conducted a paired *T*-test comparing intra-group variations in global properties and nodal attributes between pre-ICBT and post-ICBT group. The threshold for determining statistical significance was set *p* ≤ 0.05 (FDR corrected, two-tailed). GRETNA facilitated these graph-theoretic statistical analyses.

The analysis of other variables and their correlations was conducted using IBM SPSS (version 26), the normality of distributions for each variable was ascertained using the Shapiro–Wilk test. Comparative analyses between the HC group and the pre-ICBT group regarding demographic parameters (such as age, height, weight, and BMI) were conducted employing either a two-sample *T*-test or the Mann–Whitney *U* test, according to the characteristics of data distribution. Chi-square tests evaluated gender differences. Additionally, intra-group variations in clinical measurement pre-and post-ICBT group were analyzed through paired *T*-test or Wilcoxon signed-rank test. *p* ≤ 0.05 (two-tailed) was considered to indicate statistical significance. For multiple comparisons, we applied FDR correction, *p* ≤ 0.05 (FDR corrected, two-tailed) was considered as statistically significant difference.

**Figure 1:**
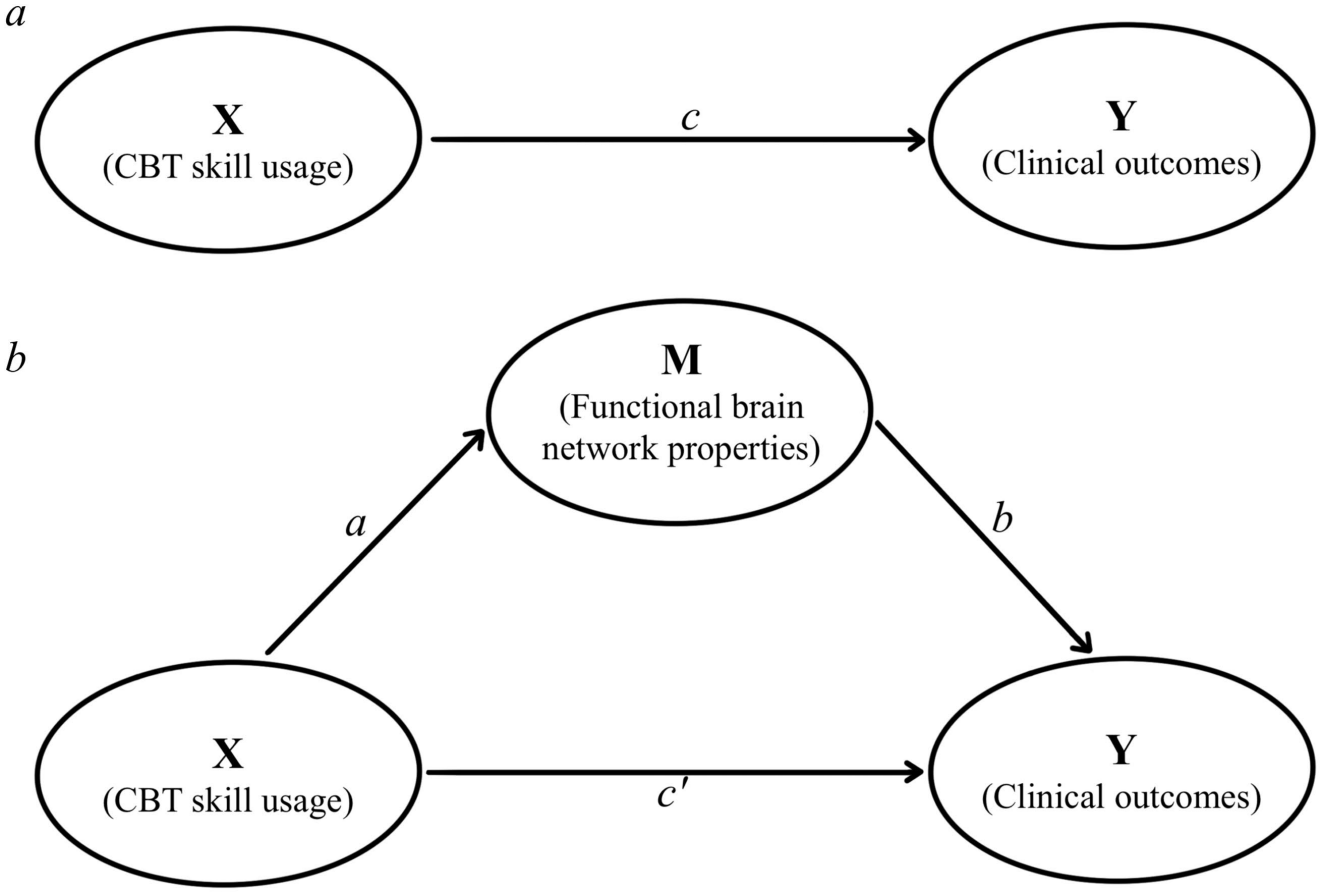
The mediation analyze model applied for this study. **(a)** The Path e represents the total effect of the independent variable (X) on the outcomes (Y); **(b)** the Path a represents the effect of the independent variable (X) on the mediator variable (M). Path b measures the effect of the mediator variable (M) on the dependent variable (Y). Path c’ represents the indirect effect of X on Y, adjusted for mediation by (*Μ*).

Subsequently, we employed partial correlation or Spearman correlation analyses to investigate the relationship between clinical outcomes and brain network properties. Additionally, mediation analysis using the PROCESS macro (43) of SPSS to assess the indirect effects of changed CBT skill usage (X) on clinical outcomes variation (Y) after ICBT intervention, mediated by changes in functional brain network properties (M),as shown in Figure 1.

## 3 Results

### 3.1 Primary outcomes

#### 3.1.1 Demographic characteristics and clinical outcomes

As illustrated in Table 1, there were no significant statistical differences in demographic variables between the HC group and pre-ICBT group. Furthermore, Table 2 presents the clinical measurement outcomes, including CES-D, PHQ-P, GAD-7, CBTSQ, CCTS-TR, FATS, and their respective sub-scales, both pre-and post-ICBT intervention, notably, the significant statistical differences were observed between the pre-ICBT group and post-ICBT group in all clinical outcomes except for the CBTSQ-Behavioral Activation (CBTSQ-BhA) and the FATS-Activity Scheduling (FATS-AS).

**Table 1:** Demographic variables differences between HC and pre-ICBT groups.

Variables (Mean ± SD)	HC group *n* = 24	pre-ICBT group *n* = 30	*P*
Age (months)	243.04 ± 13.91	235.07 ± 5.07	0.066
Gender (F:M)	20:4	25:5	1.000^#^
Height (m)	1.65 ± 0.07	1.62 ± 0.07	0.136
Weight (kg)	55.75 ± 7.97	58.60 ± 11.58	0.469
BMI (kg/m^2^)	20.47 ± 2.28	22.21 ± 3.71	0.109

SD, standard deviation; HC, Health Control; pre-ICBT, pre-intervention Internet-based Cognitive Behavioral Therapy; BMI, Body Mass Index. ^#^Indicate *p* value of Chi-square test.

**Table 2:** Clinical measurements differences between pre- and post-ICBT groups.

Clinical outcomes (Mean ± SD)	pre-ICBT group *n* = 30	post-ICBT group *n* = 30	*p* (FDR-Corrected)
CES-D	25.77 ± 1.19	14.97 ± 0.81	< 0.001^*^
PHQ-9	12.47 ± 2.47	8.33 ± 2.48	< 0.001^*^
GAD-7	14.03 ± 1.90	8.17 ± 1.26	< 0.001^*^
CBTSQ			
CBTSQ-CR	16.73 ± 5.47	33.40 ± 4.55	< 0.001^*^
CBTSQ-BhA	16.53 ± 4.94	16.37 ± 4.30	0.161
FATS			
FATS-CoR	3.40 ± 1.43	9.97 ± 1.52	< 0.001^*^
FATS-SoI	3.53 ± 1.57	8.27 ± 1.48	< 0.001^*^
FATS-ReB	2.63 ± 1.47	3.63 ± 1.22	0.002^*^
FATS-AiS	3.03 ± 1.56	3.30 ± 1.12	0.092
FATS-TS	12.60 ± 3.10	25.17 ± 2.85	< 0.001^*^
CCTS-TR			
CCTS-TR-AT	5.57 ± 2.05	14.73 ± 3.02	< 0.001^*^
CCTS-TR-BA	5.67 ± 1.90	7.73 ± 2.50	< 0.001^*^
CCTS-TR-CB	6.00 ± 1.80	11.80 ± 3.85	< 0.001^*^

SD, standard deviation; pre/post ICBT, pre/post-intervention Internet-based Cognitive Behavioral Therapy; CES-D, Center for Epidemiological Studies Depression Scale; PHQ-9, Patient Health Questionnaire-9; GAD-7, Generalized Anxiety Disorder-7.CBTSQ, Cognitive Behavioral Therapy Skills Questionnaire; CR, Cognitive Restructuring; BhA, Behavioral Activation.FATS, Frequency of Actions and Thoughts Scale; CoR, Cognitive Restructuring; SoI, Social Interaction; ReB, Rewarding Behaviors; AiS, Activity Scheduling; TS, Total Score; CCTS-TR, Competencies of Cognitive Therapy Scale –Therapist Report version; AT, Automatic Thoughts; BA, Behavioral Activation; CB, Core Beliefs. *Represented significant statistical differences at level < 0.05 (two-tailed).

#### 3.1.2 Globally functional brain network results

**Figure 2:**
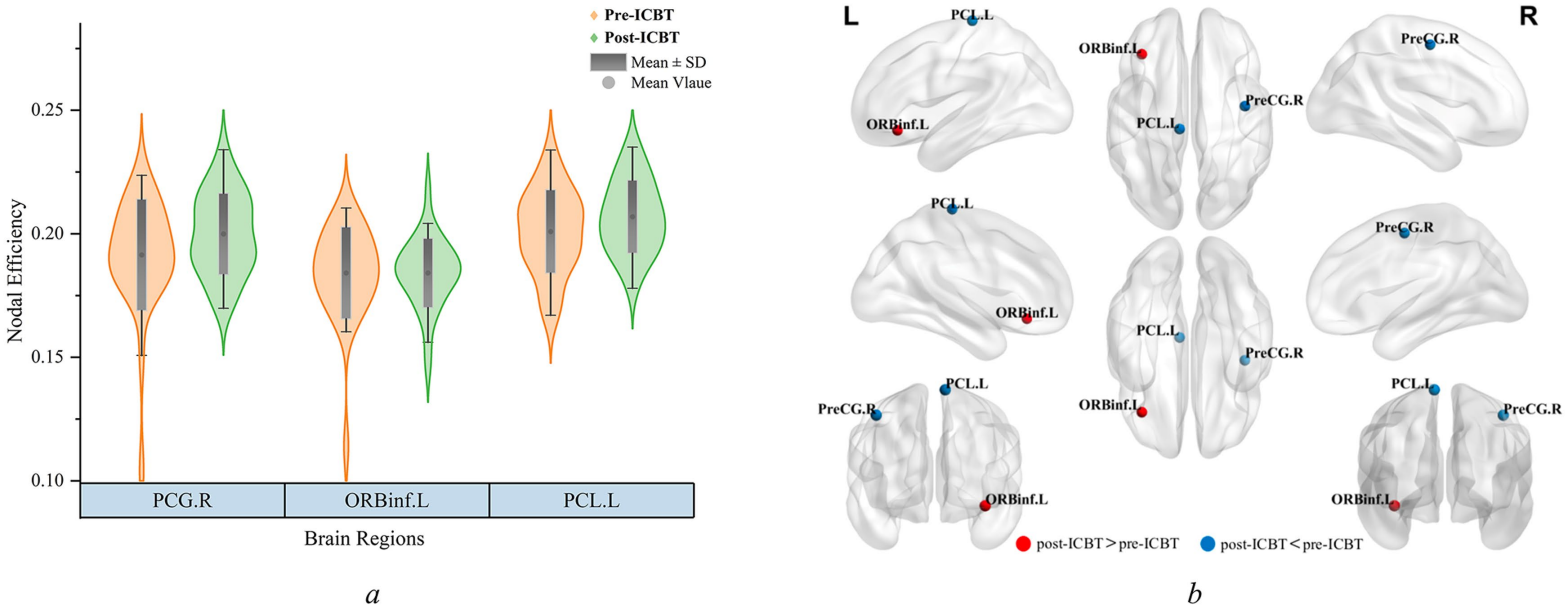
**(a,b)** Regions of significant nodal efficiency differences between the pre-ICBT group and post-ICBT group. **(b)** The red and blue balls represent increased and decreased nodal efficiencies in post-ICBT group with efficiency compared to pre-ICBT group. Pre/post-ICBT, pre/post-intervention Internet-based Cognitive Behavioral Therapy; PCL.L, left paracentral lobule; PCG.R, right precentral gyrus; ORBinf.L, orbital part of the left inferior frontal gyrus.

In our study, initially, we compared the global network properties (including Cp, Lp, *γ*, *λ*, *σ*, Eg, and Eloc) among the pre-ICBT, post-ICBT, and HC groups. As shown in Table 3, no significant differences were identified in the global topological features (*P*_FDR_ ≥ 0.05). Subsequently, nodal properties analysis revealed that after the ICBT intervention, the post-ICBT group demonstrated a significant increased *E*nod in the orbital part of the left inferior frontal gyrus (ORBinf.L; *P*_FDR_ ≤ 0.05). Additionally, significant decreases in *E*nod were noted in the left paracentral lobule (PCL.L; *P*_FDR_ ≤ 0.05) and the right precentral gyrus (PCG.R; *P*_FDR_ ≤ 0.05), as detailed in Table 4 and Figure 2. These findings indicate that ICBT can promote brain network reorganization in patients with sD by strengthening or weakening connections between certain nodes, thereby altering the topological characteristics of the brain network in patients with sD.

**Table 3:** Group comparisons of global properties AUC: pre-ICBT vs. HC, post-ICBT vs. HC, and pre- vs. post-ICBT.

Global Properties	Group	Mean ± SD	*T* value	*P* value
aEg	pre-ICBT vs. HC	0.13 ± 0.01/0.12 ± 0.01	−0.31	0.76
	post-ICBT vs. HC	0.13 ± 0.01/0.12 ± 0.01	−0.41	0.69
	pre- vs. post-ICBT	0.13 ± 0.01/0.13 ± 0.01	−0.17	0.87
aEloc	pre-ICBT vs. HC	0.18 ± 0.01/0.18 ± 0.01	−0.69	0.49
	post-ICBT vs. HC	0.18 ± 0.01/0.18 ± 0.01	−1.15	0.26
	pre- vs. post-ICBT	0.18 ± 0.01/0.18 ± 0.01	−0.51	0.62
aCp	pre-ICBT vs. HC	0.15 ± 0.01/0.14 ± 0.01	−0.75	0.46
	post-ICBT vs. HC	0.15 ± 0.01/0.14 ± 0.01	−1.52	0.14
	pre- vs. post-ICBT	0.15 ± 0.01/0.15 ± 0.01	−1.10	0.28
aγ	pre-ICBT vs. HC	0.43 ± 0.07/0.44 ± 0.08	0.22	0.83
	post-ICBT vs. HC	0.44 ± 0.07/0.44 ± 0.08	−0.05	0.96
	pre- vs. post-ICBT	0.43 ± 0.07/0.44 ± 0.07	−0.28	0.78
aλ	pre-ICBT vs. HC	0.27 ± 0.01/0.27 ± 0.02	0.21	0.84
	post-ICBT vs. HC	0.27 ± 0.01/0.27 ± 0.02	0.11	0.91
	pre- vs. post-ICBT	0.27 ± 0.01/0.27 ± 0.01	−0.13	0.90
aLp	pre-ICBT vs. HC	0.47 ± 0.02/0.47 ± 0.04	0.35	0.73
	post-ICBT vs. HC	0.47 ± 0.03/0.47 ± 0.04	0.39	0.70
	pre- vs. post-ICBT	0.47 ± 0.02/0.47 ± 0.03	0.09	0.93
aσ	pre-ICBT vs. HC	0.38 ± 0.07/0.39 ± 0.08	0.22	0.83
	post-ICBT vs. HC	0.39 ± 0.07/0.39 ± 0.08	0.07	0.95
	pre- vs. post-ICBT	0.38 ± 0.07/0.39 ± 0.07	−0.17	0.87

pre/post ICBT, pre/post-intervention Internet-based Cognitive Behavioral Therapy; AUC, area under the curve; HC, healthy controls; aEg, AUC of global efficiency; aEloc, AUC of local efficiency; aCp, AUC of clustering coefficient; aγ, AUC of normalized clustering coefficient; aλ, AUC of normalized characteristic path length; aLp, AUC of shortest path length; aσ, AUC of small-worldness.

**Table 4:** Brain regions with significantly different nodal-efficiency in the post-ICBT group compared with the pre-ICBT group.

Group	Brain regions	MNI coordinates			FDR q values
		X	Y	Z	
post-ICBT > pre-ICBT	ORBinf.L	35.98	30.71	12.11	<0.05
post-ICBT < pre-ICBT	PCG.R	41.37	8.21	52.09	<0.05
	PCL.L	−7.63	−25.36	70.07	<0.05

PCG.R, Right precentral gyrus; ORBinf.L, Orbital part of left inferior frontal gyrus; PCL.L, Left paracentral lobule.

### 3.2 Secondary outcomes

#### 3.2.1 Correlations between *E*nod and clinical outcomes for depression and anxiety

**Figure 3:**
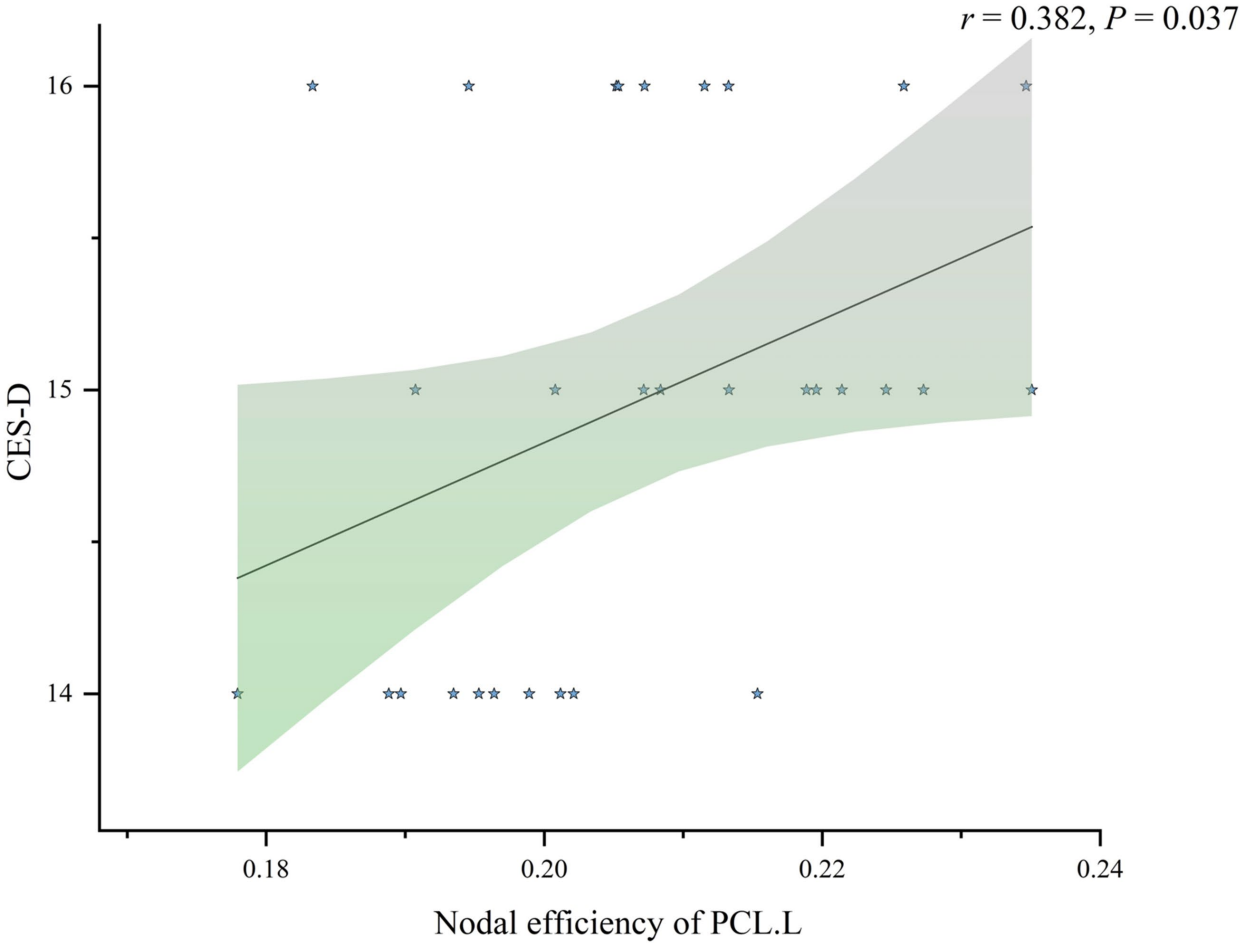
Association analysis between CES-D and nodal efficiency of PCL.L in post-ICBT group. CES-D, Center for Epidemiological Studies Depression Scale; PCLL, left paracentral lobule; post-ICBT, post-intervention Internet-based Cognitive Behavioral Therapy.

We analyzed the associations between brain network properties and clinical symptoms in patients with sD. Specifically, we examined the correlations between *E*nod in brain regions showing differences before and after ICBT intervention with scores on the CES-D, PHQ-9, and GAD-7 scales, In the pre-ICBT group, no significant correlations were observed between *E*nod in brain regions and clinical outcome measures. However, as depicted in Figure 3, a positive correlation was identified in the post-ICBT group between *E*nod in PCL.L and post-intervention CES-D scores (*r* = 0.382, *p* = 0.037).

Then we analyzed the correlations between the changes in *E*nod (post-ICBT minus pre-ICBT) across various brain regions and changes in CES-D, PHQ-9, and GAD-7 scores (post-ICBT minus pre-ICBT), the results indicated no significant associations between the variations in *E*nod and the changes in clinical outcome measures.

#### 3.2.2 Associations between *E*nod and CBT skills usage

**Figure 4:**
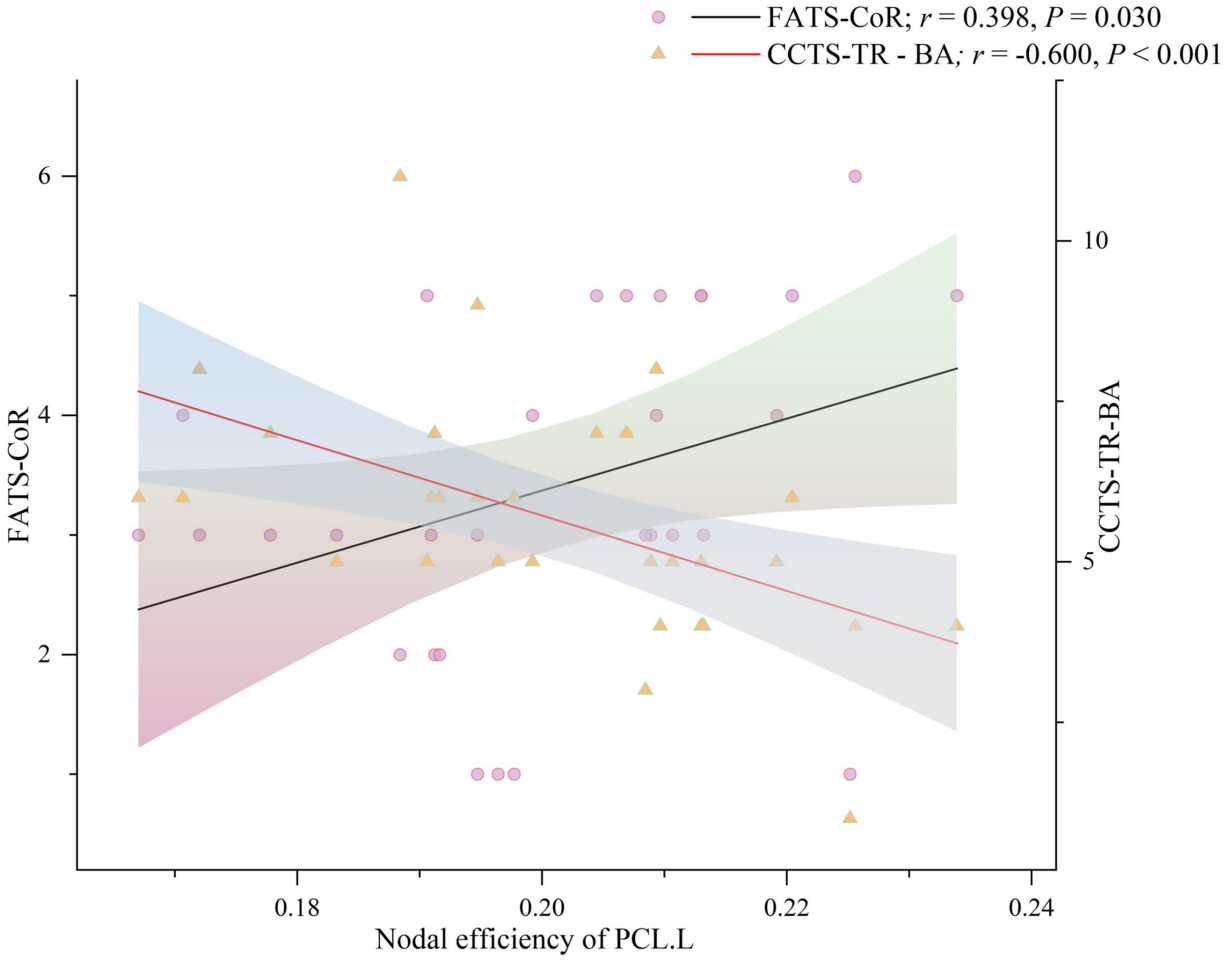
Association analysis among FATS-CoR, CCTS-TR-BA and nodal efficiency of PCL.L. in pre-ICBT group. FATS-CoR, Cognitive Restructuring in Frequency of Actions and Thoughts Scale; CCTS-TR-BA, Behavioral Activation in Competencies of Cognitive Therapy Scale Therapist Report version; PCL.L., left paracentral lobule; pre-ICBT, pre-intervention Internet-based Cognitive Behavioral Therapy.

**Figure 5:**
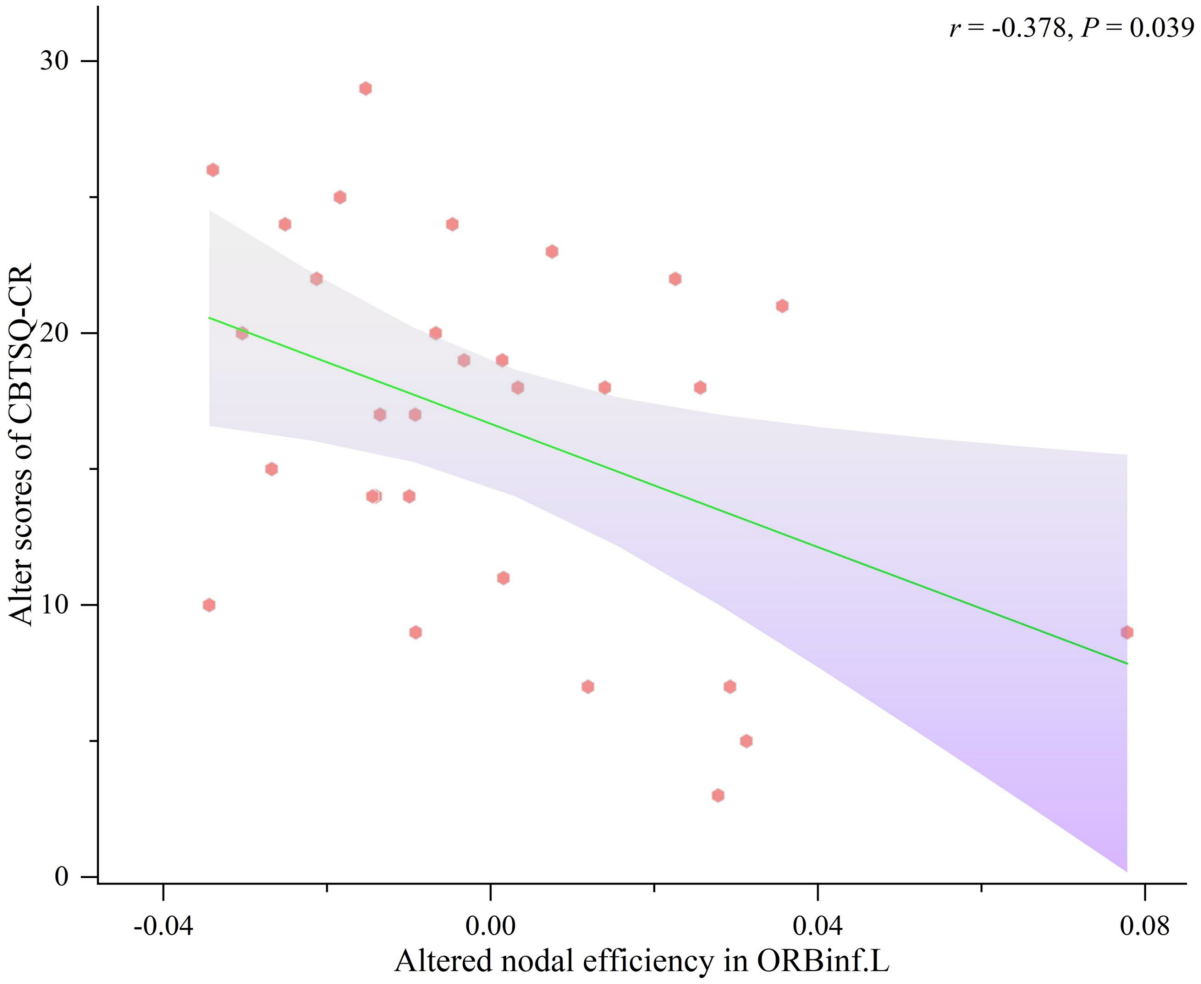
Correlation between changed scores of CBTSQ-CR and altered nodal efficiency in ORBinf.L. CBTSQ-CR, Cognitive restructuring of Cognitive Behavioral Therapy Skills Questionnaire, ORBinf.L orbital part of the left inferior frontal gyrus.

A similar correlation analysis was conducted to explore associations between brain network dynamics and CBT skills usage before and after ICBT intervention. As shown in Figure 4, in the pre-ICBT group, *E*nod in the PCL.L was positively correlated with FATS-cognitive restructuring (FATS-CoR; *r* = 0.398, *p* = 0.030) and negatively with CCTS-TR - behavioral activation (CCTS-TR-BA; *r* = −0.600, *p* < 0.001). Further analyses assessed the correlations between changes in *E*nod and variations in CBTSQ, FATS, and CCTS-TR scores. Findings indicated a negative correlation between changes in *E*nod within the ORBinf.L and CBTSQ-cognitive restructuring (CBTSQ-CR; *r* = −0.378, *p* = 0.039), detailed in Figure 5.

#### 3.2.3 Mediation analysis results

Building on previous correlation analyses in this study, changes in *E*nod within PCL.L and ORBinf.L were used as mediators to assess the indirect effects of CBT skills on clinical outcomes, as demonstrated in Table 5, reveal that modifications in brain network properties do not mediate the effects of CBT skills on clinical measures.

**Table 5:** Mediation analysis of clinical measures through the usage of CBT skills mediated by the changes in the properties of functional brain networks.

Mediator variable (changed nodal efficiency in brain regions)	Independent variable (CBT skill usage)	Outcome (changed clinical measures)	Total effect (Path *c*)	Path *a*	Path *b*	Path *c’*	Indirect effect (Path a*b)	SE of indirect effect	95% CI of indirect effects	
									Lower	Upper
PCL.L	FATS-CoR	CES-D	0.1067	0.0000	−6.3394	0.1064	0.0003	0.0225	−0.0295	0.0659
		PHQ-9	−0.5106	0.0000	29.2098	−0.5092	−0.0014	0.0534	−0.1031	0.1327
		GAD-7	0.0879	0.0000	−38.9926	0.0861	0.0018	0.0496	−0.1243	0.0893
	CCTS-TR-BA	CES-D	−0.0064	0.0013	−6.5023	0.0021	−0.0084	0.0327	−0.0893	0.0499
		PHQ-9	0.3969	0.0013	13.1999	0.3798	0.0171	0.0630	−0.1279	0.1420
		GAD-7	−0.1149	0.0013	−36.1171	−0.0681	−0.0468	0.0464	−0.1478	0.0403
ORBinf.L	CBTSQ-CR	CES-D	−0.0377	−0.0016	21.3888	−0.0040	−0.0337	0.0201	−0.0723	0.0035
		PHQ-9	−0.0318	−0.0016	−57.6694	−0.1228	0.0909	0.0600	−0.0008	0.2287
		GAD-7	−00344	−0.0016	−3.0733	−0.0392	0.0048	0.0394	−0.0877	0.0718

Path c represents the total effect of the independent variable (X) on the outcomes (Y), Path a represents the effect of the independent variable (X) on the mediator variable (M). Path b measures the effect of the mediator variable (M) on the dependent variable (Y). Path c’ represents the indirect effect of X on Y, adjusted for mediation by (M). CBT, Cognitive Behavioral Therapy; PCL.L, left paracentral lobule; ORBinf.L, orbital part of left inferior frontal gyrus; FATS-CoR, Frequency of Actions and Thoughts Scale- Cognitive Restructuring; CCTS-TR-BA, Competencies of Cognitive Therapy Scale –Therapist Report version- Behavioral Activation; CBTSQ-CR, Cognitive Behavioral Therapy Skills Questionnaire- Cognitive Restructuring; CES-D, Center for Epidemiological Studies Depression Scale; PHQ-9, Patient Health Questionnaire-9, GAD-7, Generalized Anxiety Disorder-7.

## 4 Discussion

This research primarily focused on exploring neurofunctional alterations in undergraduates with sD in China, specifically assessing the impact of ICBT on their brain network connectivity. Our study integrates neuroimaging with clinical psychometrics to assess the specific neurofunctional impacts of ICBT, particularly in a demographic that is increasingly at risk for depressive disorders but less likely to seek traditional forms of mental health care (21). Furthermore, this study provides a neuroimaging basis for the digital-network-based cognitive therapy approach.

Initially, the results indicated a significant reduction in clinical measurement scores across various measures (CES-D, PHQ-9, GAD-7) and enhanced proficiency in CBT skills from post-ICBT to pre-ICBT, suggesting that ICBT interventions can effectively alleviate the clinical symptoms of sD, moreover, trained CBT skill usage could improve the prognosis for sD patients (37, 38, 44). These findings support the efficacy of ICBT in alleviating depressive symptoms and improving mental health outcomes in young adults, even reducing the risk of suicide among school students (45), highlighting its effectiveness in enhancing emotional regulation capabilities essential for managing depressive symptoms, which were consistent with other ICBT research (46, 47).

In contrast to initial expectations, there were no significant neurofunctional disparities between pre-ICBT, post-ICBT participants and the matched HC group. Previous studies have reported no significant differences in brain function between the pre-treatment ICBT group and healthy controls in patients with depression, suggesting that neural network reorganization may not yet have emerged in certain individuals prior to ICBT intervention, potentially reflecting heterogeneity in disease progression and compensatory mechanisms (48–50). In addition, another neuroimaging study on female university students with sD utilized seed-based functional connectivity analysis with seeds in right inferior frontal gyrus (IFG), right middle frontal gyrus (MFG), and the left precentral or postcentral cortex, the study indicated no significant differences in functional connectivity between the attentional bias modification (ABM) group and the active control (AC) group before and after training (44). Another short-term fMRI study focusing on patients with mild depression identified a post-training reduction in the amplitude of low-frequency fluctuations (ALFF) exclusively in the right MFG (45). That suggests subtle functional connectivity alterations in sD even mild depression may be not detected by conventional fMRI analysis.

After the ICBT intervention, increased *E*nod was observed in the ORBinf.L, along with a negative correlation between altered *E*nod in ORBinf.L and the altered CBTSQ-CR score. Previous studies reported that OBRinf.L was a crucial language center responsible for semantic and syntactic processing (51, 52), several researches highlights that impairments in language functions, such as aphasia or reduced desire to communicate, are crucial factors contributing to depression (53, 54). In our study, following ICBT intervention, increased *E*nod in the ORBinf.L and a negative correlation between altered *E*nod in ORBinf.L and the altered CBTSQ-CR score were observed, suggesting restructuring of brain network that potentially improve language processing and thereby affect communication and may not directly enhance the cognitive restruction in sD patients. Extensive prior researches have consistently shown alterations in the brain function of the limbic system (e.g. orbitofrontal cortex and amygdala) in patients with MDD (55, 56). Although this study did not directly detect changes in the neural network of the limbic system in subthreshold depression, previous studies have substantiated the significant role of the functional network connectivity between the inferior frontal gyrus (IFG) and the orbitofrontal cortex (OFC) in emotion regulation and decision-making (57–60). Furthermore, the left inferior frontal gyrus is critical for emotional regulation (61), and emotional dyscontrol is a significant characteristic of depression. Accordingly, the observed reorganization of nodal properties in the inferior frontal gyrus in subthreshold depression may represent an important biomarker for predicting the progression of the disorder, and indicates ICBT may alleviate depressive symptoms by simultaneously enhancing linguistic processing and emotional regulation.

Additionally, our study findings indicate a decreased *E*nod in the PCL.L and PCG.R, these regions integral to the sensorimotor network (SMN). As we know, patients with depression experience impairments in both internal and external sensory processing, such as diminished capacity to process non-verbal auditory stimuli and increased pain tolerance, along with a general slowing of psychomotor functions (62–64). Previous study reported that depression severity correlated with alteration in the sensorimotor network, specifically a reduction in exteroceptive activities and an enhancement of interoceptive processes as symptoms escalate (65). Our findings suggested that ICBT may affect the sensorimotor network by potentially inhibiting the interoceptive stimulus threshold, thereby alleviating depressive symptoms. The results enhance our understanding of the neurobiological mechanisms underlying the effects of ICBT on sD.

Correlation analysis revealed that, in the pre-ICBT group, the *E*nod of the PCL.L was positively correlated with FATS-CoR scores and negatively correlated with CCTS-TR-BA scores, suggesting a potential mediating role of PCL.L in the relationship between CBT skills application and clinical outcomes. However, subsequent mediation analysis did not support this hypothesis, indicating the need for further large-scale studies to validate this assumption. In the post-ICBT group, a positive correlation between *E*nod of PCL.L and CES-D scores was observed, suggesting that reduced PCL.L network activity was associated with symptom alleviation. Although this study could not identify additional brain region changes associated with clinical measures. However, larger-scale studies in the future may provide more meaningful insights and further elucidate these relationships. They may provide valuable insights into the neural mechanisms underlying ICBT and offer a foundation for further exploration of its effects on brain function in sD patients. Moreover, these results may inform refinements in ICBT interventions, ultimately enhancing their efficacy in improving clinical outcomes and prognosis for individuals with sD.

This study suggests that ICBT may alleviate clinical symptoms in sD by modulating emotion regulation regions, the SMN, and cognitive-motor pathways. Previous studies have verified the left and right prefrontal cortices play distinct roles in emotional processing, with the left hemisphere associated with positive emotions and the right hemisphere primarily involved in negative emotions (66). Previous research has reported altered functional connectivity between ORBinf.L, PCL.L, and the limbic system in depression (55, 60, 67). Our findings revealed changes in Enod within the ORBinf.L, PCL.L, and PGC.R, suggesting that ICBT primarily enhances positive emotion regulation to improve clinical symptoms, while its effect on negative emotion processing is less pronounced. This provides further insight into the mechanisms through which ICBT modulates neural networks to alleviate symptoms in patients with sD.

### 4.1 Limitation

Despite this study revealing the impact of ICBT on brain function, this study presents several limitations. First, the research was conducted with a restricted sample size and focused exclusively on Chinese university students, which may restrict the applicability of the findings, future research is planned to broaden the sample size to further explore the impact of ICBT on changes in brain activities and assess the comparative advantages of ICBT over traditional CBT approaches. Moreover, this study did not examine the differential effects of CBT and ICBT on brain function, an aspect that should be addressed in future research to enhance the comprehensiveness of these findings. Furthermore, the specific ICBT features utilized in this study might not capture the entire range of internet-based CBT interventions, limiting the generalizability to other ICBT formats. Additionally, the HC group in this study underwent MRI scanning only at baseline, which may limit the ability to assess potential longitudinal changes in brain network properties among healthy individuals, future studies should consider incorporating longitudinal assessments of healthy controls to better delineate the specific effects of ICBT on brain networks in sD patients. Finally, the short-term of this four-week longitudinal study primarily captures the immediate neurofunctional changes following ICBT. To investigate the enduring neurobiological impacts of ICBT, in the future, extended longitudinal studies are necessary.

## 5 Conclusion

Our study investigated the neurofunctional changes in patients with sD after ICBT, highlighting significant alterations in brain activity during the resting state that correlate with clinical symptoms. Notably, it demonstrated alterations in the brain network properties of brain regions including the ORBinf.L, the PCL.L, and the PCG.R after ICBT intervention, reflecting the impact of ICBT on the language system, emotional regulation network, and the SMN. Moreover, correlations between *E*nod in ORBinf.L and PCL.L with clinical symptoms and CBT skill utilization were identified, illustrating the intricate relationships between brain network modifications and clinical outcomes. Overall, this research provides valuable insights into the neurofunctional effects of ICBT on patients with sD, offering pathways for further research and clinical application.

## Data Availability

The original contributions presented in the study are included in the article/supplementary material, further inquiries can be directed to the corresponding author.

## Footnotes

^1^ http://www.restfmri.net/forum/restplus

^2^ http://restfmri.net/forum/DPARSF

^3^ http://www.nitrc.org/projects/GRETNA
